# Estimating prevalence and burden of major disorders of the brain in Nepal: cultural, geographic, logistic and philosophical issues of methodology

**DOI:** 10.1186/1129-2377-15-51

**Published:** 2014-08-15

**Authors:** Ajay Risal, Kedar Manandhar, Timothy J Steiner, Are Holen, Rajendra Koju, Mattias Linde

**Affiliations:** 1 Department of Neuroscience, Norwegian University of Science and Technology, Trondheim, Norway; 2 Dhulikhel Hospital, Kathmandu University Hospital, Kavre, Dhulikhel, Nepal; 3 Division of Brain Sciences, Imperial College London, London, UK; 4 Pain Unit, St Olavs University Hospital, Trondheim, Norway; 5 Norwegian National Headache Centre, St Olavs University Hospital, Trondheim, Norway

**Keywords:** Nepal, Burden of disease, Population-based survey, Headache, Anxiety, Depression, Global campaign against headache

## Abstract

**Background:**

Headache, anxiety and depression are major disorders of the brain in terms of their prevalence and the burdens and costs they impose on society. Nationwide population-based studies of these disorders are necessary to inform health policy but, in research-naïve and resource-poor countries such as Nepal, a host of methodological problems are encountered: cultural, geographic, logistic and philosophical.

**Methods:**

Expert consensus was sought among researchers from different professional and cultural backgrounds in planning and conceptualizing an epidemiological study and adapting established methods to the special situation and circumstances of Nepal.

**Results:**

The methodological problems were sorted into different themes: study design; climate; geography, access and transport; sociocultural issues; safety of interviewers. Each of these was dealt with separately, and their inter-relationships explored, in finding solutions that were sometimes pragmatic. A cross-sectional questionnaire-based study, with teams of interviewers visiting households across the three physiographic divisions (with extremes in altitude) in each of the five development regions of the country, would enable national sampling with sociocultural representativeness. However, the study instruments and interviews would be in Nepali only. Transport and access challenges were considerable, and their solutions combined travel by air, bus, river and foot, with allowances for rain-damaged roads, collapsed bridges and cancelled scheduled flights. The monsoon would render many routes impassable, and therefore set an absolute time limitation. Engaging participants willingly in the enquiry would be the key to success, and several tactics would be employed to enhance the success of this, most importantly enlisting the support of local community volunteers in each study site.

**Conclusion:**

Anticipating problems in advance of investing substantial resources in a large nationwide epidemiological study in Nepal was a sensible precaution. The difficulties could be resolved or circumvented without expected compromise in scientific quality. Expert consensus was an effective means of achieving this outcome.

## Background

Headache, anxiety and depression are the major disorders of the brain (MDBs) in terms of their prevalence and the burdens and costs they impose on society [1,2].

Headache disorders are often lifelong, affecting people of either sex and any age, prevalent in every part of the world and causing disability on both personal and societal levels [3-5]. Migraine, tension-type headache (TTH) and medication-overuse headache (MOH) are, by far, the three headache disorders of greatest public-health importance [6,7]. In the Global Burden of Disease survey 2010 (GBD2010), TTH and migraine were the second and third most prevalent disorders worldwide [8], while migraine was the seventh-highest specific cause of years of life lost to disability (YLDs) [9]. Migraine accounted for more than half of all YLDs attributed to neurological disorders, and for one third of disability-adjusted life years (DALYs) in 21 regions of the world [8,10]. MOH, highly burdensome at individual level [2], may be seen in almost all cases as a complication of either migraine or TTH [6]. It has yet to be recognized in global surveys such as GBD [8-10] because of lack of epidemiological data, itself in part due to difficulties over case definition and ascertainment in population surveys [11,12].

Depression and anxiety are respectively the second and sixth leading specific causes of global YLDs [8]. Like headache disorders, these have been better studied in high-income countries, where depression is projected to become the foremost cause of DALYs by 2030 [13]. Every fourth person in Western societies is likely to suffer from anxiety disorders at some point in their lives [14].

It is clear that each of these disorders has huge public-health impact, and the collective burden from MDBs is enormous. Well-conducted methodologically-sound population-based studies are required to know what this burden is – whom it affects, in what manner, to what extent and with what consequences – and to assess need for health care, develop policy and allocate resources to meet it. Most studies of this kind have been carried out in Western Europe and North America [1,2,5]. Accordingly, the prevalence of headache has until recently been very poorly described in many large and populous regions, including South East Asia, one of the World Health Organization’s six world regions [5,15]. Mental health disorders generally are accorded low priority in low-income countries [16]. All this is despite compelling evidence that these disorders are no less burdensome in these countries. GBD2010 found that depression, migraine and anxiety were the third, fifth and sixth leading causes of disability in South Asia [8]. Extrapolating GBD2010 data to Nepal, a country in South Asia, puts depression and migraine among the top five causes of YLDs, and anxiety among the top 20 causes of DALYs [17]. Local and regional studies in Nepal have found headache to be one of the “major physical complaints” in clinic populations (both psychiatric and non-psychiatric) [18,19], but no nationwide or even regional population-based studies of any of these brain disorders have yet been conducted in this country.

*Lifting The Burden* (LTB), a UK-registered charity conducting the Global Campaign against Headache in official relations with the World Health Organization, has meanwhile been undertaking nationwide population-based studies of the burden of headache in many countries of varying income levels, and has developed methodological guidelines [20] and a survey instrument [21]. This experience may be fundamental in planning and undertaking a study in Nepal. Nevertheless, there are organizational challenges of a different order arising not only from resource limitations and impoverished infrastructure but also from the extraordinary geographical variation, unique climatic exigencies and wide sociocultural diversity of the country.

**Figure 1:**
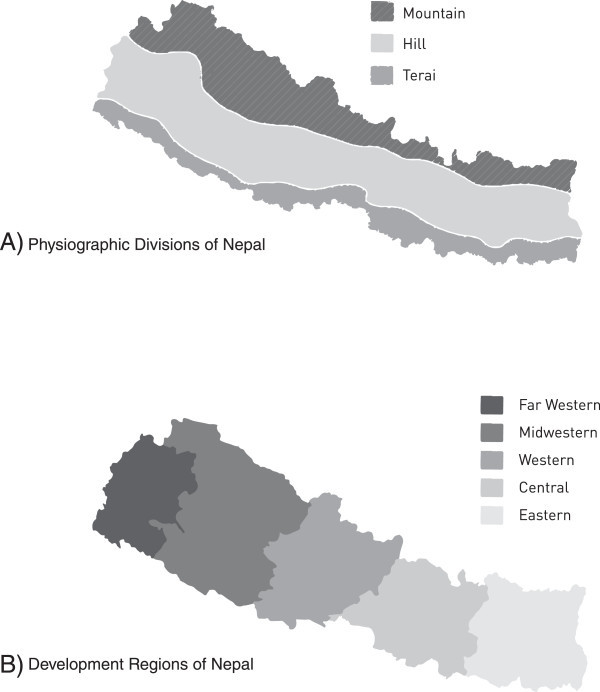
Map of Nepal showing A) the three physiographic divisions and B) the five development regions.

Nepal is among the poorest nations in the world [22], with few resources in health care, and a high illiteracy rate [23,24]. It is a small landlocked country, about 800 Km long west to east and 200 Km north to south, between the two largest (most populous) countries of the world: India and China [23]. The country is distributed North to South into three altitude-based physiographic divisions (Figure 1A) and East to West into five administrative development regions (Figure 1B), posing various logistic difficulties with regard to transport, access and accommodation [23]. More than 100 indigenous languages create potential communication barriers [25]. Distinctive and characteristic annual weather fluctuations include the monsoon, upon the arrival of which many routes across the country become immediately impassable, with no alternatives [26].

Here we identify these challenges, and describe our way of solving or circumventing them prior to investing resources in the study itself.

## Methods

A group of experts (the authors) assembled from relevant professional and diverse sociocultural backgrounds. The first meeting took place in Nepal in March 2012.

The Nepali researchers (AR, KM and RK) presented their local knowledge of topography, climate and sociocultural context. ML and TJS brought expertise in headache disorders, AR and AH in psychiatry and KM in epidemiology. TJS contributed experience from similar nationwide studies in other countries [27-30], which had led to the development of methodological guidelines [20].

Selecting and reaching a socioculturally representative sample of the adult national Nepali population, and engaging them in willing and responsive enquiry using well-chosen, relevant and appropriately adapted survey instruments, were the principal goals. Initial freely-ranging discussions (“brain-storming”) brought forward a wide range of issues likely to confront us in achieving these objectives. These were reviewed, first to discard those of limited importance or relevance (item reduction), then to bring together any that were essentially similar (refinement), and finally to sort survivors into themes. During this phase, AR, KM and ML undertook a field-visit to a nearby hill village to gain experience of the community way-of-life and household setting.

Regular email communications led to a final list of issues to be resolved. Through face-to-face discussions at further meetings in Nepal in February/March 2013 and April/May 2013, solutions to these were agreed by consensus.

## Results

Issues that would require resolution before the study could commence fell into five themes: 1. Study design; 2. Climate; 3. Geography, access and transport; 4. Sociocultural issues; 5. Safety of interviewers.

### Study design

We agreed the objective required a cross-sectional study of a nationally representative population sample, particularly with regard to sociocultural diversity. In a country with poor telephone coverage, this could be achieved only by knocking on doors and conducting face-to-face interviews [20], which would entail visiting households throughout the country. Many other issues arose from this conclusion.

Households in Nepal are scattered throughout topographically varied areas, with difficult transport links and diverse socio-cultural living conditions (see Geography, access and transport). It would be very desirable to contact all participants at the same time of year under similar weather conditions; that is, within a limited period of time (see Climate). Therefore, we would need to recruit a large number of interviewers from all over the country, and divide these into teams which could be mobilised to the different study-sites for data collection simultaneously. This decision brought out further issues related to logistics (see below).

### Climate

Nepal’s climate is characterized by heavy monsoons between June and September, bringing 250–450 mm of rainfall each month to most parts of the country [26,31]. It is the key determinant of Nepali people’s daily working schedules. To carry out the project during the monsoon would be impossible, especially in the Hill and Mountain divisions. On the other hand, in the winter season post-monsoon, most Mountain houses would be empty and locked, their occupants coming down to lower levels.

The decision to recruit many interviewers and deploy them in teams across the country would allow us to undertake data collection simultaneously in several districts, with a view to completing the survey within a month (during May).

### Geography, access and transport

**Figure 2:**
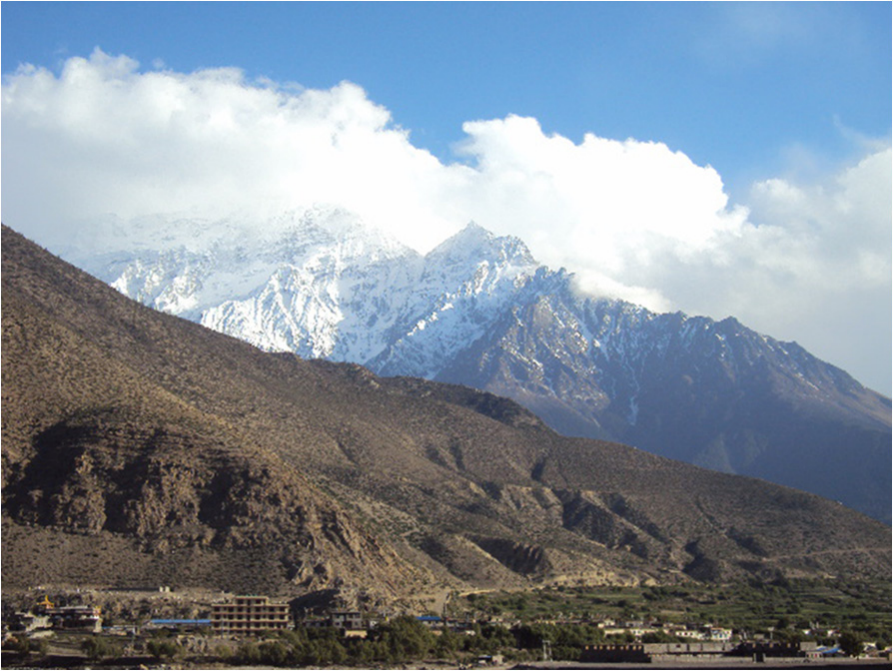
A selected Mountain district (Mustang) in the Western development region.

**Figure 3:**
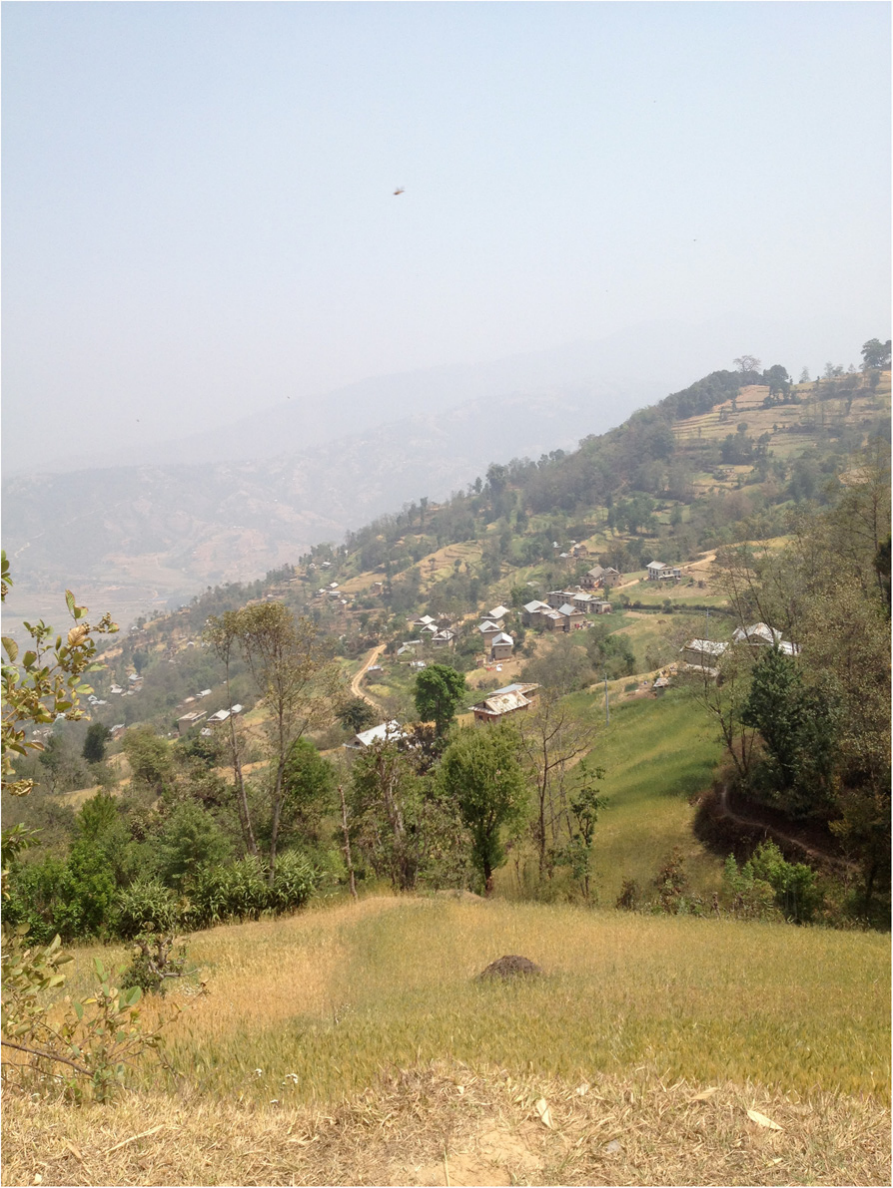
Households in a selected Hill district.

**Figure 4:**
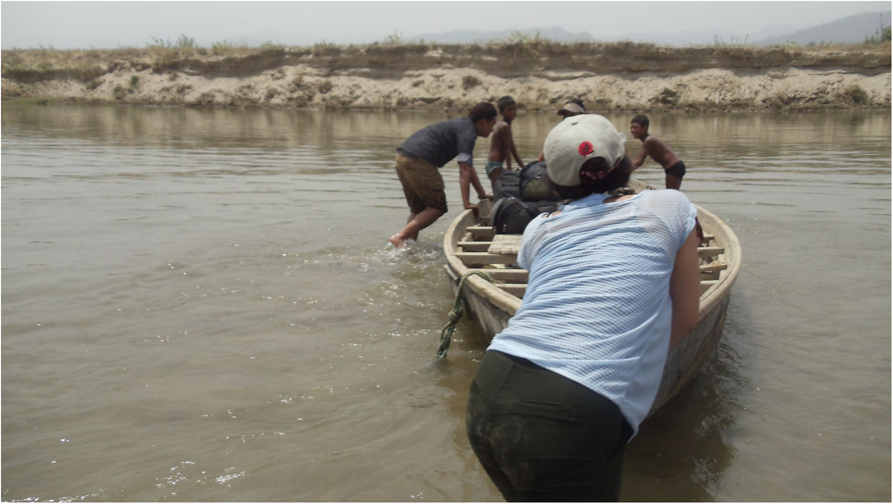
Access to a selected Terai district (Dang) in the Mid-Western development region is by boat.

**Figure 5:**
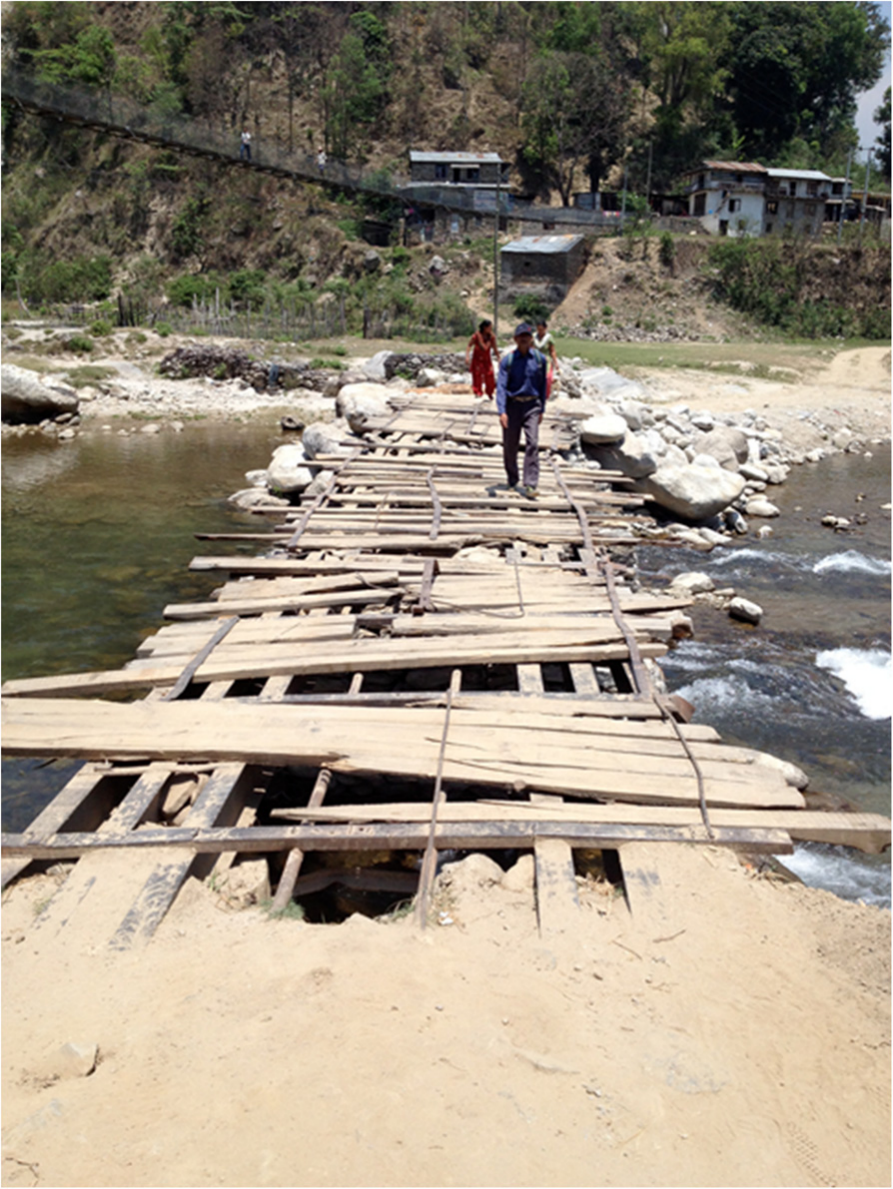
Access to a selected Mountain district (Sindhupalchok) in the Central development region is by bridge.

Nepal’s three physiographic divisions (Mountain, Hill and Terai) (Figure 1A) display enormously diverse topography: altitude ranges from below 100 m in the Terai, whereas the mountainous (Himalayan) north has eight of the world's ten tallest mountains, including Everest, and more than 240 peaks over 6,000 m [23]. With this comes steep terrain and remoteness – both major challenges for conducting a household survey. Some villages in the Mid-Western and Far-Western regions remain isolated by very poor transport links, and more so by floods and landslides during the monsoons [26].We agreed we should select one district in each of the five development regions in all three physiographic divisions to ensure national representation. This would result in 15 localities, from which survey sites would be selected in a modified cluster-sampling procedure. The challenge then was to get teams of 5–6 young people, with supplies and equipment, safely, economically and as quickly as possible to each of these 15 sites, accommodate them there for the several days of the survey, and bring them back. Not every site presented difficulties, but several did (Figures 2, 3), and called for meticulous preparation, and sometimes ingenuity, combining travel by air, bus, river (Figure 4) and foot. Allowances were necessary for rain-damaged roads, collapsed bridges (Figure 5) and (commonly) cancelled scheduled flights. The local member(s) of each team would guide the entire team (see sociocultural issues). The high likelihood of unforeseen complications meant a reserve team of interviewers should be kept available.

**Figure 6:**
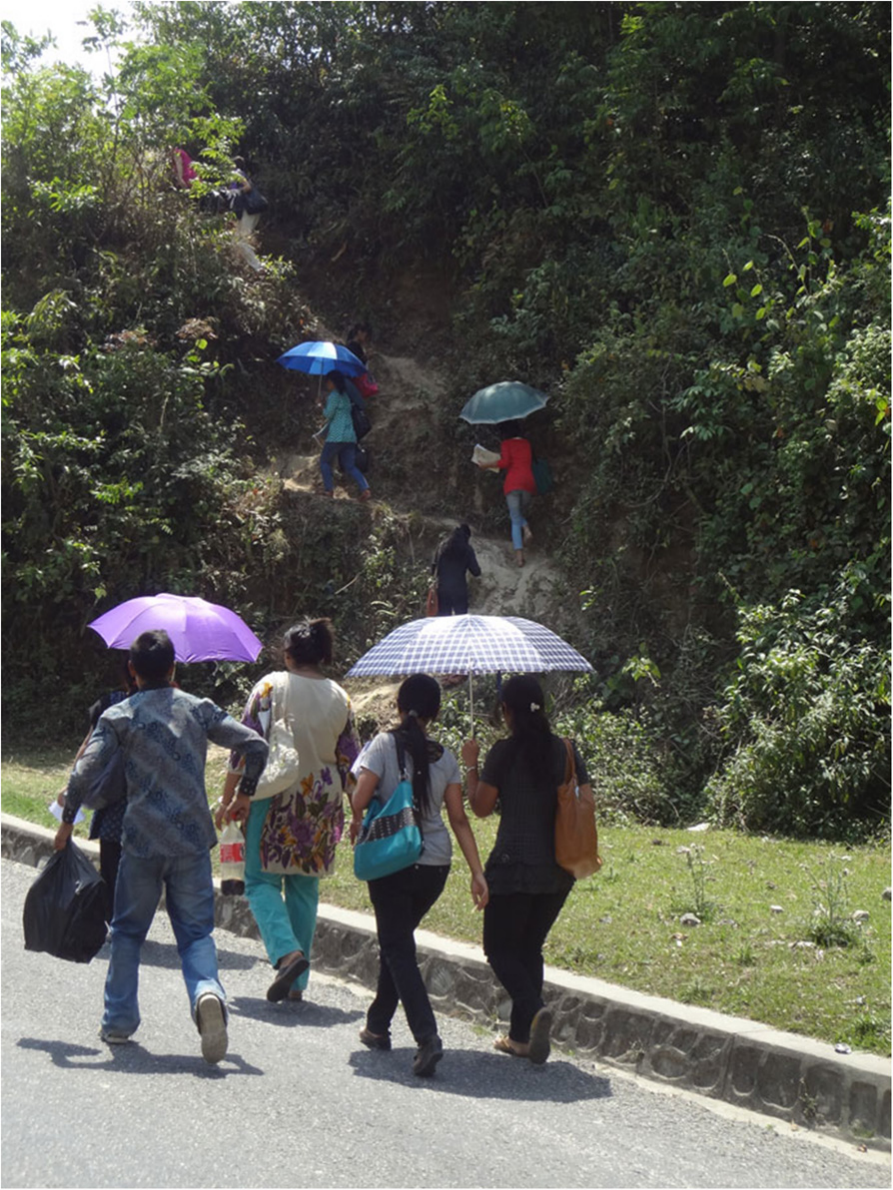
The journey to households in a selected Mountain district (Sindhupalchok) in the Central development region ends on foot and steeply uphill.

Three of the five Mountain districts were inaccessible by land. Even after flying to the nearest airports, two of these would require long uphill walks (Figure 6) to reach the selected study sites. In a Mountain district in the Far-western development region, access to the selected village would necessitate crossing a river by manually-driven cable car, then travel via India.

All five Hill districts were partly accessible by bus, with an average walk over tough terrain of 3 hours. One in the Mid-western region would require almost 2 days’ walking, mostly through the jungle. Porters would have to be hired for these long distances, since back-packs must contain altimeters, sphygmomanometers, weighing machines and questionnaires as well as personal belongings.

Access to the five Terai districts would present comparatively minor problems unless heavy rains came (which would have made a river impassable in the Mid-western region).

### Sociocultural issues

We attached importance to the following sociocultural aspects and considered whether there were necessary modifications to our study protocol or questionnaires:

a) language

b) sociocultural diversity and sensitivity

c) socioeconomic status (SES)

d) behaviour and habits

### Language

Nepal is home to more than 100 indigenous languages [25], which might appear an insuperable barrier to a population-based survey. However, while fewer than half (44.6%) of people denote Nepali as their mother tongue [25], almost everyone understands it, making Nepali the *lingua franca* of the country. Hence, we decided we would translate the questionnaire only into Nepali and use this language in interviews. Recent population-based national surveys in Nepal adopted a similar protocol [25,32,33].

### Sociocultural diversity and sensitivity

The three physiographic divisions and five development regions are ethnoculturally disparate; people might not open their doors to an unknown and socioculturally different interviewer, and equally might not consent to be interviewed. This required recruiting interviewers with a fairly representative participation from all parts of the country, aiming to have at least one member in a team from the same district, or same physiographic division and development region, accustomed to the local culture.

Most importantly of all, prior to arrival at a survey site we would enlist local support, especially from the highly-respected Female Community Health Volunteers (FCHVs). These are local women working in each ward of the Village Development Committee (VDC) and Municipality in a variety of key public-health programmes, including family planning, maternal and child health, vaccination, vitamin A supplementation, de-worming, *etc.*[34]. We agreed also that, if the interviewers resided within the selected site during the entire data-collection period, ideally in local households, rapport within these small communities would be much more firmly established.

We considered it might be difficult to interview participants privately. Most Nepalis would not feel secure away from their families while responding to queries, but might not mind doing so with the family present. Additionally, female participants might feel uncomfortable with male interviewers, and *vice versa*. These were easily solved. Potential sensitivity to enquiries into personal aspects of life required awareness, and adaptation of questions that might be found offensive; for example, in Nepal it could not be asked whether a couple were living together without being married; neither were questions permissible to unmarried individuals about the effects of headache on family planning and sex life.

### Socioeconomic status (SES)

It is difficult to assess SES among Nepalis. Income is not a good guide to financial well-being in a context of abundant self-produced resources and agricultural products in households, and consumption and income are consequently poorly correlated [32,35]. Enquiry would need to include a valuation of the annual household consumption, the monetary value of the household expenditure in the last month, and the monetary value of the household consumption of self-produced items in the last month.

The other factors traditionally contributing to SES would be similarly problematic. Questions on education have limited meaning in a country where most people are never at school [35], and in some regions would best be replaced by questions simply on literacy, with a tolerant view of the meaning of “literate” [25,32]. Occupation could be captured according to the codes and explanation in the Nepal Standard Occupational Classification [36], based on the International Standard Classification of Occupation [37].

The fact that not all households might have a chair available [32,33] required modified instructions for taking blood pressures.

### Behaviour and habits

We would add questions to reflect local behaviours and habits that might affect health or reflect use of health-care resources in this country [38]: use of home-made/herbal medications and treatments from faith-healers and ayurvedic doctors, use of tobacco (including through the traditional waterpipe or *hukka*), alcohol and cannabis [33,39,40], and use of a tumpline (*naamlo)*, a traditional head strap for carrying heavy loads, common among rural housewives and male labourers and porters [41].

### Safety of interviewers

Being in places far from home could leave the interviewers vulnerable to the dangers of assault and violence; the female interviewers in particular might be at risk. We would keep the numbers of male and female members balanced in each team, so that females would not need to walk alone during the survey. In addition, the local administration (VDC/municipality or Ward Offices) would be informed of the survey, and the whereabouts of the interviewers.

The Terai districts especially would involve risks of mosquito and sand-fly bites, and vector-borne diseases. Mosquito nets and the repellent ointments would be necessary for those travelling to these districts, as would essential medications and first-aid measures for all interviewers while travelling in the fields.

All the team leaders would have telephone contact with the Nepali researchers in case of difficulties requiring action.

## Discussion

Our endeavour is an example of a collective ambition to establish research in a new area in a developing country with scare resources and deficient research capacity and experience. MDBs, common conditions with high disability burdens in global studies, remain unexplored in Nepal with no reason to suppose they are less prevalent or have less impact on population health in this country. Their investigation is not possible without collaboration and the support of the international research community. With these, and preserving the philosophical concerns of social value, scientific validity and responsiveness, we attempted this difficult joint venture.

In most countries of the Western world, with adequate resources and few operational inconveniences [1,2], issues like illiteracy, ethnocultural variations and linguistic diversity are not factors requiring methodological adjustments. Similar studies to this in India [28] and Pakistan [29] have necessitated procedural modifications because of similar sociocultural issues. What is unique about Nepal is the extreme diversity of these, coupled with the equally extreme and highly challenging geoclimatic variation, with the attendant difficulties of transport and access. Through preparation and forethought, we attempted to anticipate the logistic and methodological problems, and sought to resolve them through a mix of ingenuity and some pragmatism but without scientific compromise.

We could design the study and modify our methodology in order to ensure national representation. We could employ large numbers of interviewers and deploy them simultaneously to all selected localities before the onset of the monsoon. Through well-planned transport arrangements and determination, we could overcome the geographic and climatic hindrances and arrive at the selected study sites. Through adaptations to the survey instruments, we could ask questions appropriate to our enquiry. But, unless the local people would be willing to participate, the study would not be done.

Hence, success of our project would depend not so much on our ability to reach different distant places, climbing mountains and crossing rivers on the way; neither would it succeed upon the excellence of our questionnaires. The achievement of this study in this country with its remote villages and isolated people would rely solely on how successfully and responsively *engaged* the participants were, on how closely the interviewers could bond with the local communities, on how strong was their rapport with the households and families, and how well they could communicate. We could not expect them to be warmly received as a group of strangers arriving unannounced, yet, unless they connected with the local community, were seen as being their own people, we would struggle with our objective. In this regard, respect for the community values, culture, tradition and social practices took on a new significance; an importance that went beyond avoidance of causing offence (which of course was itself of utmost importance).

Three of our tactics were selected with this purpose directly in mind. First, we would ensure there would be at least one local member in each team of interviewers, to introduce and guide the others. They would not only facilitate access to the site and to households, but also help in finding lodgings in the community. Second, staying with local people, in their homes, would enhance the feeling of ownership of our project by what would mostly be very small communities, bringing to the project some of the characteristics of anthropological research. Third, involvement of the FCHVs would be expected to bring many benefits: they would provide details about the selected area and, as persons of respect in the community, join and guide the data collection teams and assist in communication in the door-to-door survey.

As to compromise, we should not need to do so in the sampling procedure, and this is important. Still, language and communication might be a limitation in our study, since we made the decision to use a single Nepali version of the questionnaire throughout the country; comprehension might not always be optimal, notwithstanding our efforts using local interviewers and FCHVs. The complications of introducing and attempting to validate the instruments in even one more language (*eg*, *Maithili*) would be substantial, and since there are more than 100 others [25], the decision not to do so appears sound.

## Conclusion

Anticipating problems was a necessary precaution in advance of investing substantial resources in a large nationwide epidemiological study in Nepal. The nature of the country made many problems likely. We became aware that the principal barriers to a study would not be the very real difficulties in reaching parts of this mountainous country, with its tough terrain and unreliable transport, but the potential barriers to communication between interviewers and prospective participants, which would be less linguistic (in a country with more than 100 languages) than sociocultural. All these difficulties could be resolved methodologically, or circumvented by forethought and planning, without expected compromise in scientific quality. Expert consensus was an effective means of achieving this outcome.

## Abbreviations

DALY: Disability-adjusted life year; FCHV: Female community health volunteer; GBD: Global burden of disease; KUSMS: Kathmandu University School of Medical Sciences; LTB: Lifting the burden; MOH: Medication-overuse headache; MDB: Major disorders of the brain; NTNU: Norwegian University of Science and Technology; SES: Socio-economic status; TTH: Tension-type headache; VDC: Village Development Committee; YLD: Year of life lost to disability.

## Competing interest

TJS is a Director and Trustee of Lifting The Burden.

## Authors’ contribution

AR, KM, TS, AH, RK and ML: Conception and design. AR, KM, TS, ML: Acquisition of data. AR, KM, TS and ML: Analysis and interpretation of data. AR: Drafting the article. AR, KM, TS, AH, RK and ML: Revising it critically for important intellectual content. KM, TS, AH, RK and ML: Giving final approval of the version to be submitted.
